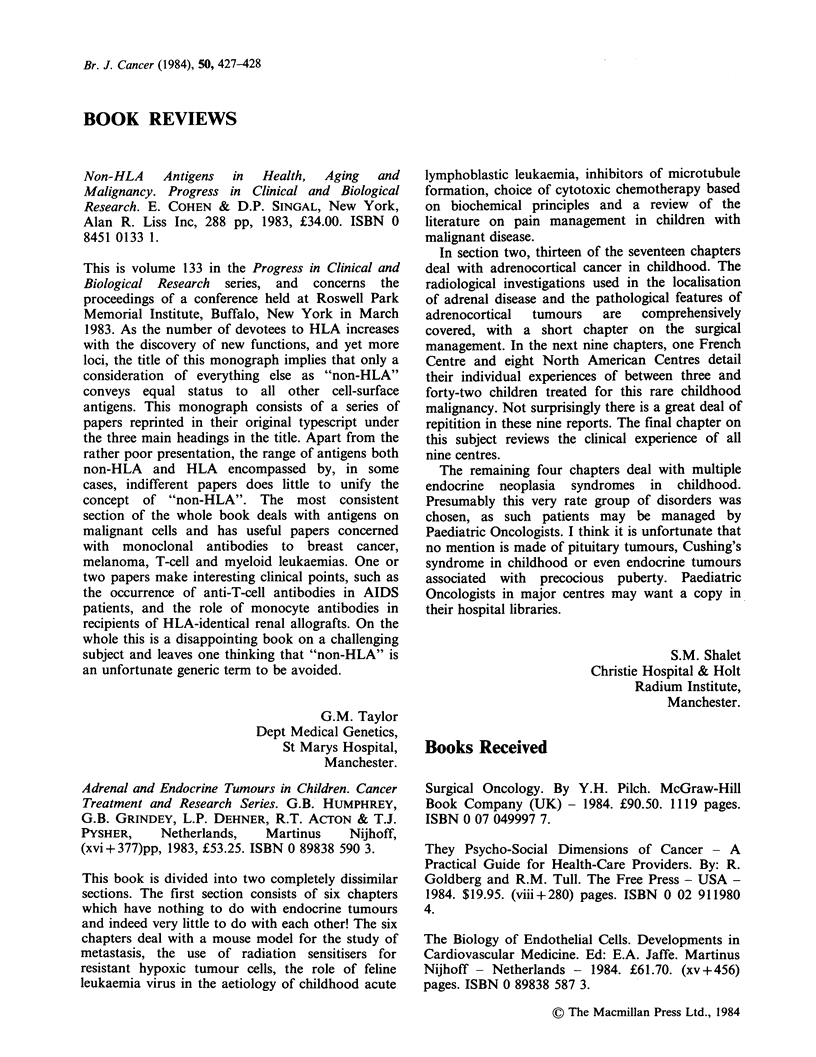# Adrenal and Endocrine Tumours in Children. Cancer Treatment and Research Series

**Published:** 1984-09

**Authors:** S.M. Shalet


*Adrenal and Endocrine Tumours in Children. Cancer Treatment and Research Series.* G.B. HUMPHREY, G.B. GRINDEY, L.P. DEHNER, R.T. ACTON & T.J. PYSHER, Netherlands, Martinus Nijhoff, (xvi+377)pp, 1983, £53.25. ISBN 0 89838 590 3.

This book is divided into two completely dissimilar sections. The first section consists of six chapters which have nothing to do with endocrine tumours and indeed very little to do with each other! The six chapters deal with a mouse model for the study of metastasis, the use of radiation sensitisers for resistant hypoxic tumour cells, the role of feline leukaemia virus in the aetiology of childhood acute lymphoblastic leukaemia, inhibitors of microtubule formation, choice of cytotoxic chemotherapy based on biochemical principles and a review of the literature on pain management in children with malignant disease.

In section two, thirteen of the seventeen chapters deal with adrenocortical cancer in childhood. The radiological investigations used in the localisation of adrenal disease and the pathological features of adrenocortical tumours are comprehensively covered, with a short chapter on the surgical management. In the next nine chapters, one French Centre and eight North American Centres detail their individual experiences of between three and forty-two children treated for this rare childhood malignancy. Not surprisingly there is a great deal of repitition in these nine reports. The final chapter on this subject reviews the clinical experience of all nine centres.

The remaining four chapters deal with multiple endocrine neoplasia syndromes in childhood. Presumably this very rate group of disorders was chosen, as such patients may be managed by Paediatric Oncologists. I think it is unfortunate that no mention is made of pituitary tumours, Cushing's syndrome in childhood or even endocrine tumours associated with precocious puberty. Paediatric Oncologists in major centres may want a copy in their hospital libraries.

S.M. Shalet
Christie Hospital & Holt
Radium Institute,
Manchester.